# Skeletal muscle interleukin 15 promotes CD8^+^ T-cell function and autoimmune myositis

**DOI:** 10.1186/s13395-015-0058-2

**Published:** 2015-09-28

**Authors:** Po-Lin Huang, Mau-Sheng Hou, Szu-Wen Wang, Chin-Ling Chang, Yae-Huei Liou, Nan-Shih Liao

**Affiliations:** Molecular Cell Biology, Taiwan International Graduate Program, Institute of Molecular Biology, Academia Sinica, and Graduate Institute of Life Sciences, National Defense Medical Center, Taipei, Taiwan; Institute of Molecular Biology, Academia Sinica, Taipei, 11529 Taiwan

**Keywords:** IL-15, Skeletal muscle, CD8^+^ T cell, Autoimmune myositis

## Abstract

**Background:**

Interleukin 15 (IL-15) is thought to be abundant in the skeletal muscle under steady state conditions based on RNA expression; however, the IL-15 RNA level may not reflect the protein level due to post-transcriptional regulation. Although exogenous protein treatment and overexpression studies indicated IL-15 functions in the skeletal muscle, how the skeletal muscle cell uses IL-15 remains unclear. In myositis patients, IL-15 protein is up-regulated in the skeletal muscle. Given the supporting role of IL-15 in CD8^+^ T-cell survival and activation and the pathogenic role of cytotoxic CD8^+^ T cells in polymyositis and inclusion-body myositis, we hypothesize that IL-15 produced by the inflamed skeletal muscle promotes myositis via CD8^+^ T cells.

**Methods:**

Expression of IL-15 and IL-15 receptors at the protein level by skeletal muscle cells were examined under steady state and cytokine stimulation conditions. The functions of IL-15 in the skeletal muscle were investigated using *Il15* knockout (*Il15*^*−/−*^) mice. The immune regulatory role of skeletal muscle IL-15 was determined by co-culturing cytokine-stimulated muscle cells and memory-like CD8^+^ T cells in vitro and by inducing autoimmune myositis in skeletal-muscle-specific *Il15*^*−/−*^ mice.

**Results:**

We found that the IL-15 protein was not expressed by skeletal muscle cells under steady state condition but induced by tumor necrosis factor alpha (TNF-α) and interferon gamma (IFN-γ) stimulation and expressed as IL-15/IL-15 receptor alpha (IL-15Rα) complex. Skeletal muscle cells expressed a scanty amount of IL-15 receptor beta (IL-15Rβ) under either conditions and only responded to a high concentration of IL-15 hyperagonist, but not IL-15. Consistently, deficiency of endogenous IL-15 affected neither skeletal muscle growth nor its responses to TNF-α and IFN-γ. On the other hand, the cytokine-stimulated skeletal muscle cells presented antigen and provided IL-15 to promote the effector function of memory-like CD8^+^ T cells. Genetic ablation of *Il15* in skeletal muscle cells greatly ameliorated autoimmune myositis in mice.

**Conclusions:**

These findings together indicate that skeletal muscle IL-15 directly regulates immune effector cells but not muscle cells and thus presents a potential therapeutic target for myositis.

**Electronic supplementary material:**

The online version of this article (doi:10.1186/s13395-015-0058-2) contains supplementary material, which is available to authorized users.

## Background

Interleukin 15 (IL-15) is widely expressed as its high-affinity binding partner IL-15 receptor alpha (IL-15Rα) at the RNA level, and the expression of IL-15 is subjected to multiple post-transcriptional regulations [1]. Newly synthesized IL-15 and IL-15Rα proteins form complex in the endoplasmic reticulum [2, 3]. The complex is transported to and displayed on the cell surface and used via the IL-15 receptor beta (IL-15Rβ) and the common gamma chain (γ_c)_ expressed on neighboring cells [4, 5]. The binding of IL-15/IL-15Rα complex to IL-15Rβ/γ_c_ on the responding cells triggers the activation of JAK-STAT3/5, phosphatidylinositol 3-kinase (PI3K)-AKT, and p42/44 mitogen-activated protein kinase (ERK) signaling pathways [6, 7]. This mode of IL-15 usage termed “trans-presentation” is essential for the development and homeostasis of memory CD8^+^ T cells, CD8αα^+^ intestinal intraepithelial T cells, and natural killer (NK) cells [8]. Notably, all naturally produced IL-15 proteins detected in human and mouse serum and in activated dendritic cells are in complex with IL-15Rα [3, 9].

IL-15 has been thought of as a myokine due to the abundant mRNA expression in the skeletal muscle [10]. Moreover, expression of *Il15* mRNA is up-regulated along myoblast differentiation [11]. Previous studies showed that exogenous treatment or overexpression of IL-15 promotes myoblast differentiation and muscle hypertrophy and ameliorates muscle wasting in cancer cachexia [12–16]. Whereas skeletal-muscle-specific overexpression or systemic infusion of IL-15 induces skeletal muscle atrophy in vivo [17–19]. Moreover, recent studies showed that exercise endurance is reduced in *Il15*^*−/−*^ mice and increased in skeletal-muscle-specific *Il15*-transgenic mice [18, 20]. However, the extensor digitorum longus (EDL) and soleus muscle isolated from these two types of genetically engineered mice show similar fatigue index ex vivo [21]. Therefore, the function of IL-15 in the skeletal muscle under non-disease conditions remains elusive.

Inflammatory myopathies are a group of diseases that involve chronic muscle inflammation (myositis) accompanied with muscle weakness [22]. The three main types including polymyositis, dermatomyositis, and inclusion body myositis are classified based on distinct clinicopathological features. They are idiopathic, but an autoimmune pathogenesis is strongly implicated. Dermatomyositis is mediated by complement, while polymyositis and inclusion-body myositis is mediated by CD8^+^ T cells that target major histocompatibility complex (MHC) class-I-expressing muscle cells through secreting cytotoxic effector molecules [23–25]. Interferon alpha (IFN-α), interferon gamma (IFN-γ), tumor necrosis factor alpha (TNF-α), and IL-1α/β are up-regulated in the muscle of myositis patients, which is implicated in the mediation of Th1 and pro-inflammatory responses [26]. Stimulation of myoblasts with IL-1α/β, TNF-α, or IFN-γ induces IL-15 production in vitro [27, 28]. Consistently, elevation of IL-15 protein has been observed in the skeletal muscle of myositis patients [27, 28]. Local up-regulation of IL-15 in certain autoimmune diseases positively associates with disease severity. An increase of IL-15 protein in the intestinal mucosa and synovial cavity of celiac disease and rheumatoid arthritis patients, respectively, stimulates dendritic cells, NK cells, and effector T cells to exacerbate the disease [29, 30]. However, the role of skeletal muscle IL-15 in myositis has not been reported. Given the essential role of IL-15 in memory CD8^+^ T-cell survival and function and the pathogenic role of cytotoxic CD8^+^ T cells in polymyositis and inclusion-body myositis, we hypothesize that the skeletal muscle IL-15 promotes autoreactive CD8^+^ T-cell function, which contributes to the development of autoimmune myositis.

Considering that the level of IL-15 RNA may not reflect the level of protein due to post-transcriptional regulations [31] and that the usage of IL-15 by skeletal muscle cells has not been studied, we examined the expression of IL-15 and its receptors at the protein level in skeletal muscle cells under steady state and cytokine-stimulated conditions. We then examined the function of endogenous IL-15 in the skeletal muscle cell and its role in the development of autoimmune myositis.

## Methods

### Mice

C57BL/6J, B6.Cg-Tg(ACTA1-cre)79Jme/J (human alpha-skeletal actin (*ACTA*)*-cre*), and B6.129S1-synaptotagmin VII (*Syt7*)^*tm1Nan*^/J (*Syt7*^−/−^) were purchased from The Jackson Laboratory (Bar Harbor, ME). *Il15*^*−/−*^ mice were purchased from Taconic and backcrossed to the C57BL/6J for at least 14 generations. *Il15ra*^*−/−*^ mice were developed in our laboratory and backcrossed to the C57BL/6J for 27 generations [32]. *Il15*^*flox/flox*^ (*Il15*^*f/f*^) mice were generated in our laboratory as previously described [33]. Skeletal-muscle-specific *Il15*^*−/−*^ (*ACTA-Il15*^*−/−*^) mice were generated by crossing *Il15*^*f/f*^ with *ACTA-cre* mice. All experimental procedures were performed in accordance with protocols approved by the Institutional Animal Care and Use Committee of Academia Sinica.

### Culture of skeletal muscle cells

**Fig. 1:**
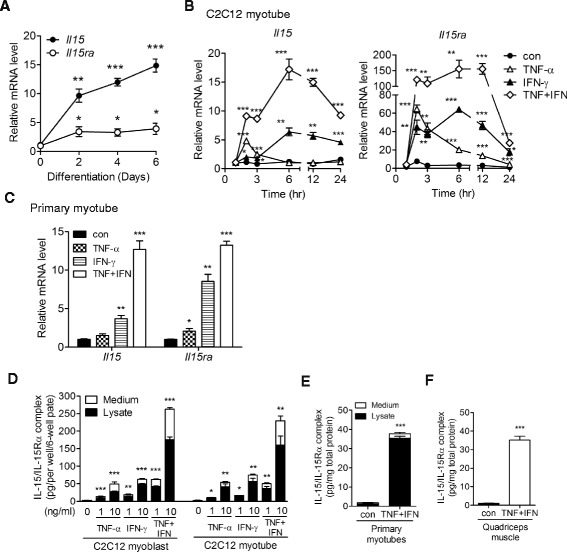
Skeletal muscle cells express IL-15/IL-15Rα protein complex in response to TNF-α and IFN-γ stimulation. **a** Expression of *Il15* and *Il15ra* mRNA during C2C12 myoblast differentiation. Samples were collected before (0) and 2, 4, and 6 days after differentiation induction. **b** Expression of *Il15* and *Il15ra* mRNA in C2C12 myotubes treated with TNF-α (10 ng/ml), IFN-γ (10 ng/ml), TNF-α + IFN-γ (TNF + IFN, 10 ng/ml each), or without cytokine (*con*) for 1, 2, 3, 6, 12, and 24 h. **c** Expression of *Il15* and *Il15ra* mRNA in primary myotubes treated with TNF-α (5 ng/ml), IFN-γ (5 ng/ml), TNF + IFN (5 ng/ml each), or without cytokine (*con*) for 24 h. **d** Expression of IL-15/IL-15Rα complex protein in C2C12 myoblasts and myotubes treated with TNF-α (1 or 10 ng/ml), IFN-γ (1 or 10 ng/ml), TNF + IFN (1 or 10 ng/ml each), or without cytokine (*con*) for 24 h. **e** Expression of IL-15/IL-15Rα complex protein in primary myotubes treated with TNF + IFN (5 ng/ml each) or vehicle (*con*) for 24 h. **f** Expression of IL-15/IL-15Rα complex protein in skeletal muscle in vivo. TNF-α plus IFN-γ (1 mg each/injection) or PBS (*con*) was injected into the quadriceps muscles of mice three times at 4-h intervals. The injected muscles were collected 16 h after the last injection. Total RNA was isolated and analyzed by qPCR (**a–c**). Protein lysate and cell culture medium were collected and measured by ELISA (**d–f**). Data in (**a–c**) were triplicates and representative of two independent experiments with similar result. Data in (**d, e**) were pooled from three independent experiments. Data in (**f**) was pooled from three mice in each group. Data are mean ± SEM. *p < 0.05, **p < 0.01, ***p < 0.001, in comparison to “0” or “con”

C2C12 myoblasts were maintained in Dulbecco’s modified Eagle’s medium (DMEM) containing 10 % fetal bovine serum (FBS). Confluent C2C12 myoblasts were shifted to differentiation medium (DMEM containing 2 % horse serum) for myotube differentiation. Unless indicated otherwise (Fig. 1a), C2C12 myotubes were used 4 days after differentiation induction, when about 80 % of culture plate surface was covered by myotubes. Primary myoblasts were isolated from the limb muscle of 1- to 3-day-old neonatal mice and purified by sorting of α7 integrin-positive cells as previously described [34]. Rat anti-α7 integrin monoclonal antibody, CA5.5, was kindly provided by Dr. Chung-Chen Yao (National Taiwan University). Purified primary myoblasts (about 25,000 cells/cm^2^) were cultured in growth medium (40 % Ham’s F-10, 40 % DMEM, 20 % FBS, 2.5 ng/ml bFGF) for 1 day and then switched to differentiation medium (DMEM containing 5 % horse serum). Some primary myoblasts already fused to form nascent myotubes during the 1-day culture in growth medium. After changing to differentiation medium, well-differentiated primary myotubes appeared in day 1 and were used for experiments in day 2.

### Measurement of IL-15/IL-15Rα complex protein

Cells or skeletal muscle tissues were homogenized in non-denaturing cell lysis buffer (Cell signaling) containing protease inhibitor cocktail (Roche). For quantification of surface IL-15Rα-bound IL-15, muscle cells were incubated with acid glycine buffer as previously described [35]. The amount of IL-15/IL-15Rα complex was measured by mouse IL-15/IL-15Rα Complex ELISA Kit (eBioscience).

### Western blotting

To study signal transduction, adherent cells were washed, and a fixed volume of sodium dodecyl sulfate (SDS) sample buffer (50 mM Tris-Cl, pH 6.8, 6 % glycerol, 0.02 % bromophenol blue, 2 % SDS, and 2 % β-mercaptoethanol) was directly added to culture wells. Immunoblotting was performed using anti-p-STAT5 and t-STAT5 antibodies (Cell signaling) following the protocol of antibody manufacturer.

### Gene expression analysis by quantitative real-time PCR (qPCR)

Total RNA was extracted by TRIzol, treated with DNase I, then reverse transcribed into cDNA by SuperScript III reverse transcriptase (Invitrogen, Life Technologies). Quantitative PCR was performed by Applied Biosystems 7500 Real-Time System using SYBR Green Master Mix. Relative gene expression levels were calculated by ABI 7500 software using acidic ribosomal phosphoprotein P0 (36B4) as the internal control. Primer pairs were pre-designed in PrimerBank [36] or designed by Primer Express software (Life Technologies) and are listed in Additional file 1: Table S1.

### Co-culture of C2C12 cells with CD8^+^ T cells

The *H-2K*^*b*^ (NM_001001892) coding sequence (nt. 77-1186) was cloned from the cDNA library of primary myotubes of C57BL/6J mice and then inserted into the EcoRI cloning site of lentiviral package plasmid pLKO AS3.1.EGFP3′. The full-length *ovalbumin* (*OVA*) cDNA with restriction enzyme cutting sites, 5′-NheI and 3′-EcoRI, was amplified by PCR from pcDNA3/OVA plasmid (kindly provided by Dr. Mi-Hua Tao, Academia Sinica) and inserted into lentiviral package plasmid pLAS2w.Ppuro. All lentiviral packaging plasmids and protocols were from the RNAi Core Facility, Academia Sinica. The EGFP-positive C2C12 myoblasts were sorted and subsequently infected with lentivirus-carrying OVA expression cassette and selected in growth medium containing 2 μg/ml puromycin (Sigma). The expression of H-2K^b^ and OVA was confirmed by flow cytometry and quantitative real-time PCR (qPCR), respectively. For CD8^+^ T cells, splenocytes of OT-1 mice were stimulated with OVA peptide as previously described [37] then cultured in medium containing IL-15 (30 ng/ml, eBioscience). After culturing for 8 days, more than 90 % of cells were CD8^+^CD44^hi^CD122^hi^, and these cells were used for experiments. C2C12 cells were treated with TNF-α and IFN-γ (1 ng/ml each) for 24 h. After washing with PBS, 24-h IL-15-deprived CD8^+^ T cells and 5 μg/ml brefeldin A (Sigma) were added simultaneously to C2C12 cells, followed by brief centrifugation to make the cell-cell contact. CD8^+^ T cells were harvested for intracellular cytokine analysis after co-culturing for 8 h.

### Flow cytometry analysis

Lymphocyte surface markers were stained with antibodies against CD19 (6D5), H57 (H57-597), CD8 (53-6.7), CD44 (IM7), CD122 (TM-b1), and NK1.1 (PK136) (eBioscience and BioLegend). C2C12 cells were stained with biotin-conjugated antibodies against IL-15 (Cat. No. 500-P173Bt, PeproTech), IL-15Rα (Cat. No. BAF551, R&D), IL-15Rβ (CD122, clone: TM-b1, eBioscience), and γ_c_ (CD132, Cat. No. 554470, BD Biosciences) then incubated with APC-conjugated streptavidin (BD Biosciences). CD8^+^ T cells were stained with fixable viable dye eFluor506 (eBioscience) then intracellularly stained with antibodies against IFN-γ (XMG1.2, eBioscience) and granzyme B (NGZB, eBioscience). All data were acquired on LSRII (BD Biosciences) and analyzed by the FlowJo (Tree Star).

### Induction of experimental autoimmune myositis

Preparation of mouse fast-type skeletal muscle myosin-binding protein C (C protein) fragment and induction of myositis were done as previously described with little modification [38]. C protein fragment purified from *Escherichia coli* lysates was washed with 60 % isopropanol solution to remove endotoxin as previously described [39]. Female mice, 8–10 weeks old, were intradermally immunized with 200 μg C protein emulsified in 200 μl complete Freund’s adjuvant (Sigma-Aldrich) at footpads, back, and tail base. Simultaneously, 0.5 μg pertussis toxin (Calbiochem) was intraperitoneally injected. The quadriceps muscle was harvested for histology and gene expression analysis 14 days after immunization. Each muscle block was cut into four sections with intervals of at least 200 μm. Histopathologic scoring was based on the most severe inflammation observed in the section among four sections and graded as previously described [38].

### Statistics

Results were represented as mean ± SEM. Statistical significance was determined by unpaired, two-tailed, Student’s *t*-test using GraphPad Prism 5 (GraphPad, San Diego, CA). *p* values less than 0.05 were considered significant.

## Results

### Skeletal muscle cells express IL-15/IL-15Rα complex protein in response to TNF-α and IFN-γ stimulation

Previous studies found up-regulation of *Il15* RNA during myoblast differentiation [11] and pro-inflammatory cytokine stimulation [27]. Given the presence of post-transcriptional regulation of IL-15 expression and that all circulating IL-15 are in complex with IL-15Rα [9], we examined the expression of IL-15/IL-15Rα complex protein by skeletal muscle cell under the two conditions mentioned above. During a 6-day C2C12 myoblast-to-myotube differentiation, the cells showed a greater than 10-fold and a 3-fold increase of *Il15* and *Il15ra* mRNA, respectively (Fig. 1a), whereas the cell lysate and culture medium contained no detectable IL-15/IL-15Rα protein by ELISA with a sensitivity of 3.9 pg/ml. This result indicates that although the level of *Il15* and *Il15ra* mRNA increased along myoblast-to-myotube differentiation, there was little production of IL-15/IL-15Rα protein. We next examined whether pro-inflammatory cytokines induce IL-15 and IL-15Rα expression by skeletal muscle cells. We found that TNF-α and IFN-γ each up-regulated *Il15* and *Il15ra* mRNA in C2C12 myotubes with distinct kinetics and together exerted a synergistic effect (Fig. 1b). A similar synergistic effect occurred in primary myotubes (Fig. 1c). TNF-α and IFN-γ treatment also up-regulated the expression of the IL-15/IL-15Rα complex protein in C2C12 myoblasts and myotubes (Fig. 1d) and in primary myotubes (Fig. 1e) in vitro. Most of the IL-15/IL-15Rα proteins were present in the C2C12 cell lysates (Fig. 1d), while nearly all IL-15/IL-15Rα proteins were present in the primary myotube lysate (Fig. 1e). Injection of TNF-α and IFN-γ into the quadriceps muscle in mice also greatly induced the expression of IL-15/IL-15Rα protein in the injected muscle (Fig. 1f).

IFN-α, another cytokine up-regulated in myositis muscle [26], induced *Il15* and *Il15ra* mRNA and IL-15/IL-15Rα complex protein to the level similar to those induced by IFN-γ or TNF-α (Additional file 2: Figure S1). Other pro-inflammatory factors, such as IL-1α, IL-1β, and LPS, only transiently up-regulated *Il15ra* or *Il15* mRNA but showed small effect on the induction of the IL-15/IL-15Rα protein (Additional file 2: Figure S1). Together, these results indicate that the expression of the IL-15/IL-15Rα complex protein by skeletal muscle cells was undetectable under steady state conditions, while induced by TNF-α, IFN-γ, or IFN-α that associates with Th1 response in myositis.

### IL-15 is present on the surface of skeletal muscle cells

**Fig. 2:**
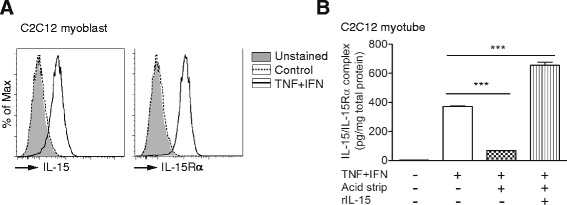
IL-15 is presented on the surface of skeletal muscle cells. **a** Expression of IL-15 and IL-15Rα on C2C12 myoblasts. Cells were treated with TNF-α and IFN-γ (TNF + IFN) or without cytokine (*Control*) for 24 h and then stained with anti-IL-15 and IL-15Rα antibodies for flow cytometry analysis. “*Unstained*” indicates cells without antibody staining. Data are representative of three independent experiments with similar results. **b** Quantification of IL-15 bound by cell surface IL-15Rα on C2C12 myotubes. C2C12 myotubes were first treated with TNF-α and IFN-γ for 24 h, incubated with acid glycine buffer to dissociate IL-15 from cell surface IL-15Rα, and then re-treated with exogenous IL-15. Cell lysates from each step were collected and the amount of IL-15/ IL-15Rα complex was measured by ELISA. Data are mean ± SEM of triplicates and representative of two independent experiments with similar results. ***p < 0.001

As the majority of the cytokine-induced IL-15/IL-15Rα complex was in the lysates of the myoblast and myotube (Fig. 1d, e), we next examined whether the complex is present on the cell surface. We found that TNF-α and IFN-γ induced expression of IL-15 and IL-15Rα on the surface of C2C12 myoblasts as detected by flow cytometry (Fig. 2a). For the cytokine-treated C2C12 myotubes, dissociation of IL-15 from IL-15Rα on the cell surface by washing cells with acidic glycine buffer resulted in an 80 % reduction of IL-15/IL-15Rα in the cell lysate (Fig. 2b), indicating that 80 % of IL-15 was presented by IL-15Rα on the cell surface. This reduction was not due to alteration of IL-15Rα by the acid treatment, as the level of IL-15/IL-15Rα resumed following the addition of exogenous IL-15 (Fig. 2b). These results indicated that the majority of the cytokine-induced IL-15 was present on the surface of skeletal muscle cells as IL-15/IL-15Rα complex.

### Skeletal muscle cells express a scanty amount of IL-15Rβ and only respond to a high concentration of IL-15 hyperagonist, but not IL-15

**Fig. 3:**
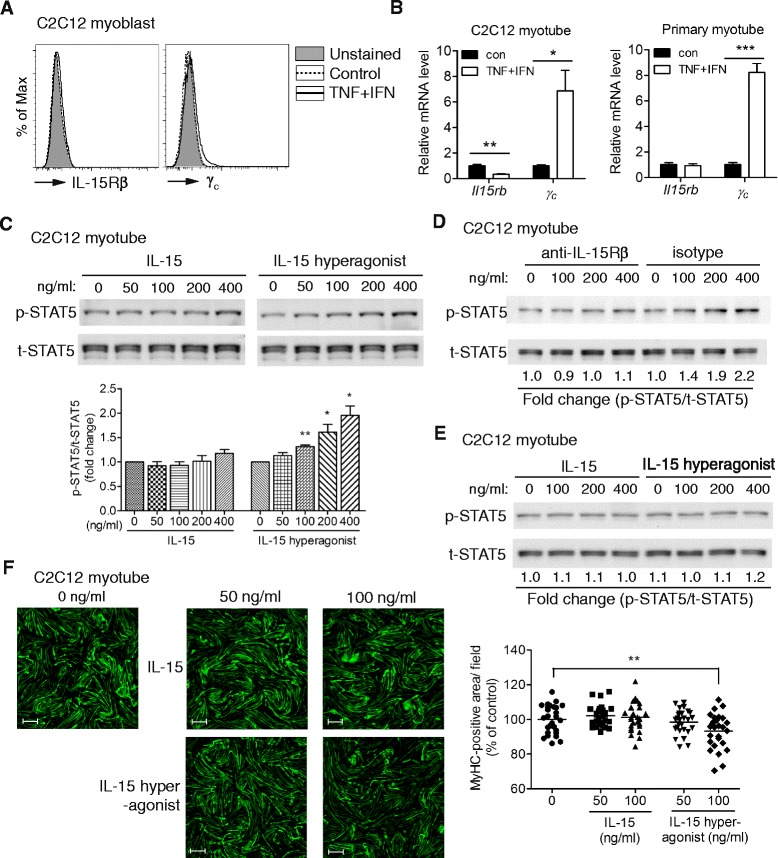
High concentration of IL-15 hyperagonist, but not IL-15, induces STAT5 signaling and atrophy in skeletal muscle cells. **a** Analysis of IL-15Rβ and γ_c_ expression on C2C12 myoblasts under the same condition of Fig. 2a. Data are representative of three independent experiments with similar results. **b** Expression of *Il15rb* and *γ*_*c*_ mRNA in C2C12 and primary myotube. Samples were collected 24 h after cytokine treatment and analyzed by qPCR. Data are triplicates and representative of two independent experiments with similar results. **c** Immunoblotting of STAT5 phosphorylation in C2C12 myotubes after treating with IL-15 or IL-15 hyperagonist for 30 min. Quantification data of four independent experiments are shown below. **d** Anti-IL-15Rβ antibody diminished IL-15 hyperagonist-induced STAT5 phosphorylation in C2C12 myotubes. Myotubes were pretreated with anti-IL-15Rβ or isotype control antibody for 1 h and then treated with IL-15 hyperagonist for 30 min. Data are representative of two independent experiments with similar results. **e** Immunoblotting of STAT5 phosphorylation in C2C12 myotubes that were first stimulated with TNF-α and IFN-γ for 24 h and then treated with exogenous IL-15 or IL-15 hyperagonist for 30 min. Data are representative of two independent experiments with similar results. **f** Accumulation of myosin heavy chain (MyHC) in C2C12 myotubes treated with IL-15 (50 and 100 ng/ml), IL-15 hyperagonist (50 and 100 ng/ml), or vehicle (0 ng/ml). After 48 h, cells were immunostained with FITC-labeled anti-myosin antibody to evaluate the accumulation of MyHC. MyHC-positive area per microscopic field are shown in the *right panel* and represented as the percentages of con. Quantification data were pooled from three independent experiments. Each *symbol* represents the quantification data from one microscopic field. Scale bar = 500 μm. Data are mean ± SEM. *p < 0.05, **p < 0.01, ***p < 0.001

Despite of various reported IL-15 functions in the skeletal muscle [12–16], the IL-15-induced signals in skeletal muscle cells remains unexplored. We first examined the expression of IL-15Rβ and γ_c_ on C2C12 myoblasts by flow cytometry and detected no IL-15Rβ expression but a low level of γ_c_ induced by TNF-α and IFN-γ treatment (Fig. 3a). The more sensitive qPCR also detected induction of *γ*_*c*_ mRNA, but not *Il15rb* mRNA, in C2C12 and primary myotubes by the cytokines (Fig. 3b). Moreover, the level of *Il15rb* mRNA in the primary myotube was 16 times lower than that in the C2C12 myotube based on the Ct value of qPCR.

Due to the possibility of a very low level of IL-15Rβ expression, we examined whether IL-15 or an IL-15 hyperagonist induces signal transduction in C2C12 myotubes. The latter is a fusion protein of IL-15 and IL-15Rα-sushi domain, which possesses higher binding affinity for IL-15Rβ/γ_c_ (*K*_*d*_ = 780 pM) and promotes a stronger proliferation of IL-15Rβ/γ_c_-bearing cells (EC_50_ = 25 pM) than IL-15 (*K*_*d*_ = 13.5 nM, EC_50_ = 3 nM) [40]. We found that IL-15 did not induce STAT5 phosphorylation at a concentration up to 400 ng/ml (26.7 nM) (Fig. 3c). Whereas the IL-15 hyperagonist induced moderate but significant STAT5 phosphorylation at concentrations of 100 ng/ml (3.4 nM) and higher (Fig. 3c), which are much higher than that required for the proliferation of IL-15Rβ/γ_c_-bearing cells (EC_50_ = 25 pM) and for STAT5 phosphorylation in pre-activated murine CD8^+^ cells (EC_50_ = 10 pM) [40, 41]. In addition to STAT5 phosphorylation, IL-15 and IL-15 hyperagonist did not induce phosphorylation of STAT3, AKT, and ERK in skeletal muscle cells (data not shown). The IL-15 hyperagonist-induced STAT5 phosphorylation was completely blocked by the IL-15Rβ-blocking antibody TM-b1 (Fig. 3d). However, neither IL-15 nor IL-15 hyperagonist induced STAT5 phosphorylation in C2C12 myotubes pre-treated with TNF-α and IFN-γ (Fig. 3e), which is in line with the decrease of *Il15rb* mRNA after cytokine treatment (Fig. 3b). Consistent with the STAT5 phosphorylation results (Fig. 3c), 100 ng/ml of IL-15 hyperagonist, but not IL-15, induced atrophy of C2C12 myotubes (Fig. 3f). In summary, C2C12 and primary skeletal muscle cells expressed a scanty IL-15Rβ under steady state condition and TNF-α/IFN-γ stimulation and only responded to a high concentration of IL-15 hyperagonist, but not IL-15. Moreover, we found that *Il15*^*−/−*^ mice showed normal skeletal muscle mass (Additional file 3: Figure S2), myoblast differentiation (Additional file 4: Figure S3), cardiotoxin-induced muscle regeneration (Additional file 5: Figure S4), and compensatory hypertrophy of plantaris muscle (Additional file 6: Figure S5). The in vitro and in vivo results collectively suggest that the skeletal muscle cells do not use IL-15.

### IL-15 deficiency does not affect the response of primary myotube to TNF-α and IFN-γ stimulation

**Fig. 4:**
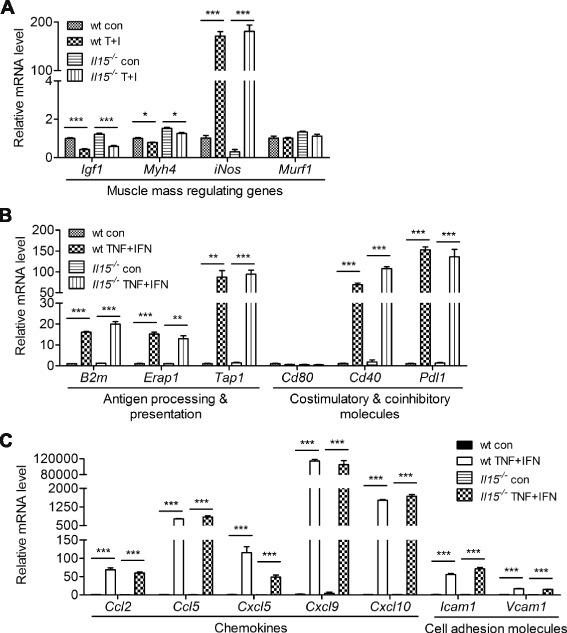
IL-15 deficiency does not affect the responses of primary myotube to TNF-α and IFN-γ stimulation. **a–c** Expression profiling of genes involved in the regulation of skeletal muscle mass and immune system in wt and *Il15* ^*−/−*^ primary myotubes. Samples were collected 24 h after TNF-α and IFN-γ treatment and analyzed by qPCR. Data are mean ± SEM of triplicates. Data are representative of two independent experiments with similar results. *p < 0.05, **p < 0.01, ***p < 0.001

As TNF-α and IFN-γ induced abundant expression of IL-15/IL-15Rα on skeletal muscle cells, the local concentration of IL-15/IL-15Rα trans-presentation may be high enough to trigger signaling through the limited number of IL-15Rβ/γ_c_ on adjacent muscle cells. We thus examined whether IL-15 affects the response of the skeletal muscle to TNF-α and IFN-γ by comparing wild type (wt) and *Il15*^*−/−*^ primary myotubes. As TNF-α and IFN-γ induce muscle wasting under cancer cachexia and IL-15 was shown to prevent it [16, 42, 43], we first examined genes that regulate muscle mass. Upon TNF-α and IFN-γ stimulation, wt and *Il15*^*−/−*^ primary myotubes showed similar reduction in the hypertrophy-related genes *Igf1* and *Myh4* and increase in the atrophy-related gene *iNos* (Fig. 4a). We next examined the immune regulatory genes affected by IL-15 in immune cells [44–48]. We found that TNF-α and IFN-γ induced the expression of immune regulatory genes in wt and *Il15*^*−/−*^ primary myotubes to similar extents, including molecules in the MHC class I antigen presentation pathway and for T-cell co-stimulation and inhibition, chemokines, and cell adhesion molecules (Fig. 4b, c). These results indicate that skeletal muscle IL-15 does not affect the expression of protein homeostasis and immune regulation genes by skeletal muscle cells in response to TNF-α and IFN-γ, which is consistent with the idea that the muscle cells do not use their own IL-15.

### Skeletal muscle cells stimulated with TNF-α and IFN-γ present antigen and provide IL-15 to memory-like CD8^+^ T cells

**Fig. 5:**
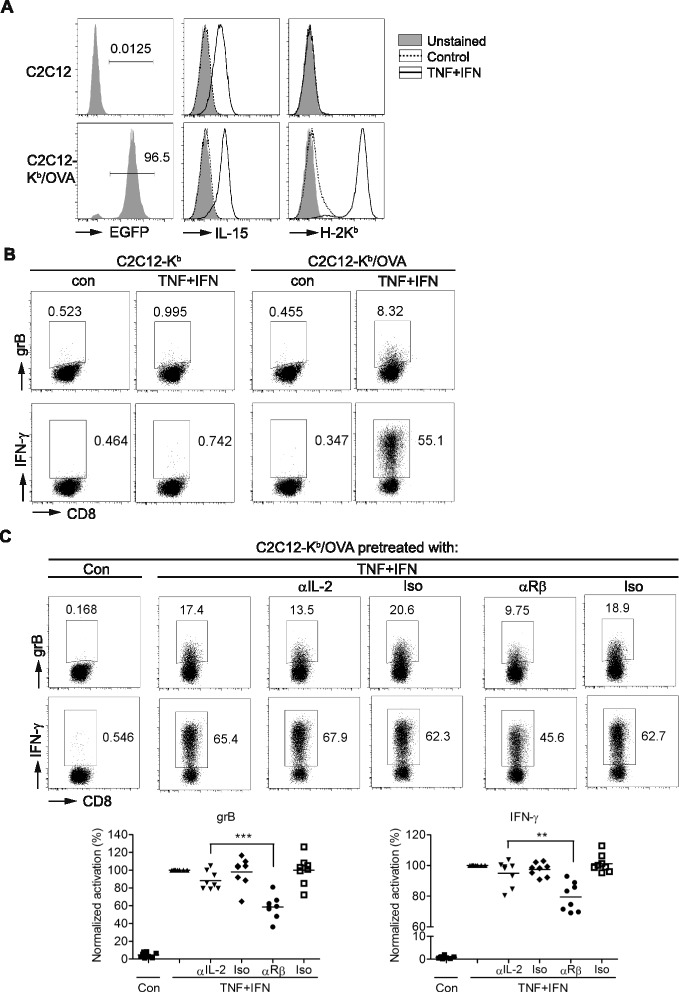
Skeletal muscle cells presented antigen and provided IL-15 to promote the effector function of CD8^+^ T cells under TNF-α and IFN-γ treatment. **a** Expression of IL-15 and H-2K^b^ on parental C2C12 and C2C12-K^b^/OVA cells. Cells were treated with TNF-α and IFN-γ (TNF + IFN) or without (*Control*) for 24 h, stained with anti-IL-15 or anti-H-2Kb antibody, then analyzed by flow cytometry. “*Unstained*” indicates C2C12 or C2C12-K^b^/OVA myoblast without antibody staining. **b** Expression of grB and IFN-γ by CD8^+^ T cells co-cultured with cytokine-treated C2C12-K^b^ or C2C12-K^b^/OVA myoblasts for 8 h. After co-culture, CD8^+^ T cells were stained with anti-grB and IFN-γ antibody by intracellular staining then analyzed by flow cytometry. Data are representative of three independent experiments with similar results. **c** Effect of anti-IL-2 (αIL-2, S4B6) or anti-IL-2/15Rβ (αRβ, TM-β1) antibody on grB and IFN-γ production by CD8^+^ T cells co-cultured with TNF + IFN-pretreated C2C12-K^b^/OVA myoblasts. The percentages of grB^+^ and IFN-γ^+^ cells in CD8^+^ T cells are indicated in the *dot plots*. Data from independent experiments were compiled as % normalized activation using the percentage of grB^+^ or IFN-γ^+^ cells in CD8^+^ T cells of the TNF + IFN-pretreated C2C12-K^b^/OVA cell co-culture group of each experiment as 100 % (*bottom panels*). Isotype controls, Rat IgG2a (Iso) and Rat IgG2b (Iso). Each *symbol* is representative of one independent experiment. **p < 0.01, ***p < 0.001

IL-15 is a well-known survival and activation factor for memory CD8^+^ T cells. As inflammatory cytokines induce the expression of IL-15/IL-15Rα and antigen presentation molecules by myoblasts and myotubes [49], we designed a muscle-cell-T-cell co-culture system to assess whether the muscle cells directly activate CD8^+^ T cells and the role of IL-15 in this process. We generated a C2C12 myoblast subline that stably expresses H-2K^b^-EGFP (C2C12-K^b^). As overexpression of H-2K^b^ impairs myoblast differentiation in this study and [50], this co-culture system is for myoblast and T cells. We then transduced full-length OVA gene into C2C12-K^b^ myoblast as an endogenous antigen and generated C2C12-K^b^/OVA myoblast. TNF-α and IFN-γ greatly induced the expression of IL-15 and H-2K^b^ on C2C12-K^b^/OVA cells (Fig. 5a). This high induction of H-2K^b^ might partly result from the up-regulation of β2-microglobulin (B2m) by the cytokines (Fig. 4b), because B2m is essential for the stabilization of MHC class I molecule in correct conformation to receive the peptide in the ER and to move from the ER to the cell surface [51]. The cytokine-stimulated C2C12-K^b^/OVA, but not C2C12-K^b^, cells induced production of granzyme B (grB) and IFN-γ by memory-like OT-1 cells (Fig. 5b). We then found that an IL-15Rβ-blocking antibody, but not IL-2-neutralizing antibody, suppressed grB and IFN-γ production by the memory-like OT-1 cells (Fig. 5c). As the cytokine-stimulated C2C12-K^b^/OVA cells were washed before co-culturing with OT-1 cells in the presence of the exocytosis inhibitor brefeldin A, IL-15 was presumably only present on the muscle cell surface. These results indicate that myoblasts stimulated with TNF-α and IFN-γ present antigen and IL-15 to memory-like CD8^+^ T cells to promote their effector function. Given that both myoblasts and myotubes function as antigen-presenting cells under inflammation, what we observed in the myoblast-CD8^+^ T-cell co-culture is likely applicable to myotubes.

### Skeletal muscle IL-15 promotes the progression of autoimmune myositis

**Fig. 6:**
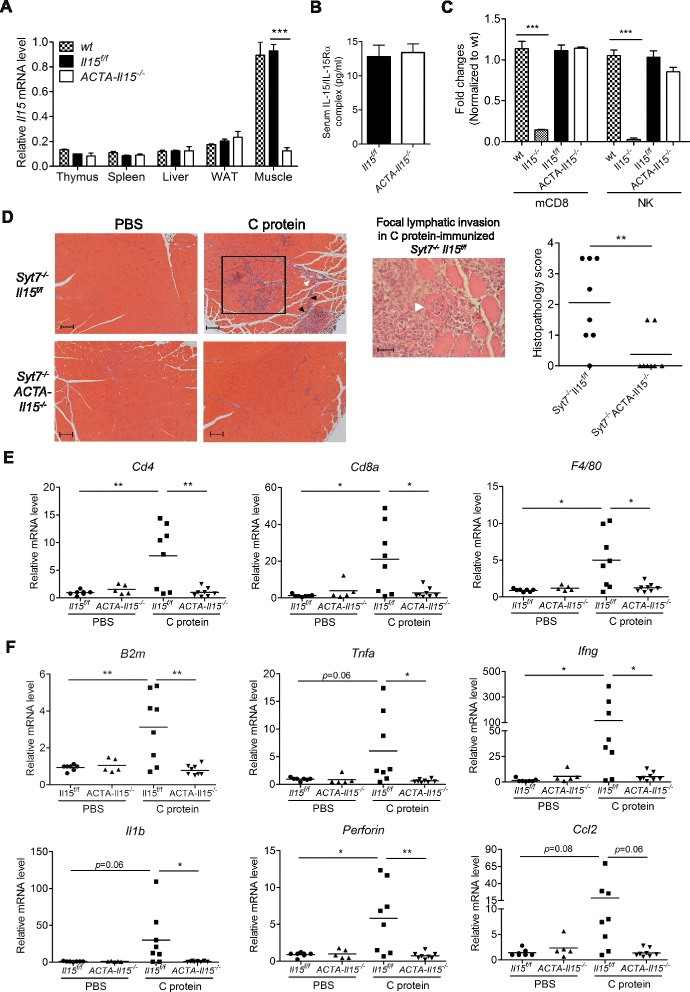
Skeletal muscle IL-15 contributes to the progression of autoimmune myositis. **a** The expression of *Il15* mRNA in various tissues of wt (*n* = 3), *Il15* ^*f/f*^ (*n* = 5), and *ACTA-Il15* ^*−/−*^ (*n* = 5) mice was detected by qPCR. *WAT* white adipose tissue. Data are mean ± SEM. ***p < 0.001. **b** The amount of IL-15/IL-15Rα complex protein in the serum of *Il15* ^*f/f*^ and *ACTA-Il15* ^*−/−*^ mice (*n* = 9–10) was measured by ELISA. Data are mean ± SEM. **c** Comparison of memory CD8^+^ T cell (mCD8) and NK cell (NK) level in the peripheral blood among wt, *Il15* ^*−/−*^, *Il15* ^*f/f*^, and *ACTA-Il15* ^*−/−*^ mice (*n* = 5 in each group). Fold change was calculated by normalizing the percentage of indicated cell type in mutant mice to that in wt mice. mCD8 were H57^+^CD19^−^CD8^+^CD44^hi^CD122^hi^, and NK were H57^−^CD19^−^NK1.1^+^. Data are mean ± SEM. ***p < 0.001. **d** Mononuclear cell infiltration in the quadriceps muscles of *Syt7* ^*−/−*^ *Il15* ^*f/f*^ and *Syt7* ^*−/−*^ *ACTA-Il15* ^*−/−*^ mice 14 days after C protein immunization (*Left*). Mononuclear cell infiltration was found in the endomysium (*black square*), perimysium (*black arrowhead*), and perivascular region (*white arrowhead*). Focal lymphatic invasion of myofibers were observed in C-protein-immunized *Syt7* ^*−/−*^ *ACTA-Il15* ^*−/−*^ mice as indicated by the *white arrowhead* (*Middle*). The histopathology scores were compiled in the *right panel* with each symbol representing one mouse (*Right*). Scale bar in left = 100 μm; middle = 25 μm. **p < 0.01. **e** Expression of mononuclear cell markers, *Cd4*, *Cd8*, and *F4/80*, mRNA in the quadriceps muscles of C-protein-immunized mice measured by qPCR. Each *symbol* representing one mouse. *p < 0.05, **p < 0.01. **f** Expression of immune-relevant genes mRNA in the quadriceps muscles of C-protein-immunized mice measured by qPCR. Each *symbol* representing one mouse. *p < 0.05, **p < 0.01

TNF-α and IFN-γ are commonly expressed in the skeletal muscle of patients suffering from inflammatory myopathies, in which CD8^+^ T cells infiltrate and play a critical role in disease progression [23, 25, 52]. The enhancement of memory-like CD8^+^ T-cell effector function by myoblast IL-15 in vitro prompted us to examine the role of skeletal muscle IL-15 in autoimmune myositis in vivo. We first generated skeletal-muscle-specific *Il15*^*−/−*^ mice by crossing *Il15*^*f/f*^ mice with *ACTA*-*cre* mice. The *ACTA-Il15*^*−/−*^ mice showed an 80 % reduction of *Il15* mRNA specifically in the skeletal muscle (Fig. 6a) with normal levels of IL-15/IL-15Rα complex and NK and memory CD8^+^ T cells in the peripheral blood (Fig. 6b, c).

As two previously reported autoimmune myositis models, wt mice immunized with C protein [38, 53] and *Syt7*^*−/−*^ mice [54, 55], did not develop myositis in our hand, we immunized mice of *Syt7*^*−/−*^ background with C protein because impaired muscle membrane sealing due to Syt7 deficiency facilitates myositis induction [54, 56]. Similar to previously reported pathology in autoimmune myositis, we found that C-protein-immunized *Syt7*^−/−^*Il15*^*f/f*^ mice developed mononuclear cell infiltration (Fig. 6d). The mononuclear cells predominantly infiltrated into the endomysium as well as the perimysium and perivascular region (Fig. 6d (left)). The immunized *Syt7*^−/−^*Il15*^*f/f*^ mice also developed focal lymphatic invasion of muscle fibers that features CD8^+^ T-cell-mediated myositis (Fig. 6d (middle)). Whereas C-protein-immunized *Syt7*^−/−^*ACTA*-*Il15*^*−/−*^ mice showed a significantly reduced mononuclear cell infiltration and histopathology score (Fig. 6d). We also found elevation of *Cd4*, *Cd8α*, and *F4/80* mRNA in the skeletal muscle of the immunized *Syt7*^−/−^*Il15*^*f/f*^ mice (Fig. 6e), suggesting infiltration of CD4^+^ and CD8^+^ T cells and macrophages. The expression of MHC class I subunit *B2m*; pro-inflammatory cytokines *Tnfa*, *Ifng*, and *Il1β*: and effector molecule *Prf1* were also induced in the skeletal muscle of the immunized *Syt7*^−/−^*Il15*^*f/f*^ mice (Fig. 6f). Whereas all these molecules examined were not induced in the C-protein-immunized *Syt7*^−/−^*ACTA*-*Il15*^*−/−*^ mice (Fig. 6e, f). These results together demonstrate that genetic ablation of skeletal muscle IL-15 greatly reduced the pathogenesis of autoimmune myositis.

## Discussion

In this study, we examined the expression and function of the skeletal muscle cell IL-15. We found that the IL-15/IL-15Rα protein was not produced by skeletal muscle cells under steady state conditions but highly induced by TNF-α and IFN-γ and presented on the cell surface. Rather than being used by skeletal muscle cells, the IL-15 directly promoted the effector function of memory-like CD8^+^ T cells in vitro and exacerbated the progression of autoimmune myositis in vivo. These results suggest that the endogenous IL-15 of the skeletal muscle cell functions as an immune regulator in an inflammatory skeletal muscle microenvironment.

IL-15 has been reported to affect skeletal muscle physiology, but IL-15 signaling in skeletal muscle cells remains unclear. Our findings shed some light on this. First, C2C12 and primary muscle cells expressed very low levels of *Il15rb* mRNA and undetectable levels of cell surface IL-15Rβ (Fig. 3a, b). Second, a soluble IL-15 hyperagonist, but not IL-15, induced STAT5 phosphorylation in C2C12 myotube at 100 ng/ml and above (Fig. 3c). These concentrations are much higher than that required for binding to IL-15Rβ/γ_c_ and the pg/ml level of circulating the IL-15/IL-15Rα complex [9], which suggests that the myotubes do not use soluble IL-15 or IL-15/IL-15Rα complex under steady state conditions. Consistently, our in vivo studies showed that IL-15 deficiency did not affect skeletal muscle mass, cardiotoxin-induced muscle regeneration, and compensatory hypertrophy of plantaris muscle (Additional file 3, 5, and 6: Figure S2, S4, and S5). Although trans-presentation of cell-bound IL-15/IL-15Rα among the stimulated muscle cells may reach a high enough local concentration to trigger signaling through the sparsely expressed IL-15Rβ/γc, *Il15* knockout did not affect the response of the primary myotube to TNF-α and IFN-γ in vitro (Fig. 4). Collectively, our results support that the skeletal muscle cells do not use IL-15 for skeletal muscle growth, regeneration, or inflammatory responses.

Our in vitro results appear different from previous in vitro studies showing that exogenous IL-15 promotes skeletal muscle hypertrophy [12, 14]. The difference may partly result from the intrinsic differences between the C2C12 and C2 cell lines used in this and previous studies, respectively. C2C12 cells have a higher differentiation potency and insulin-like growth factor 1 (IGF-I) level than C2 cells [57]. IGF-I is a strong stimulator for muscle hypertrophy [58]. An earlier study indicates that IL-15-induced C2 differentiation was only revealed in the absence of IGF-I signaling [13]. Therefore, it is possible that the higher level of IGF-I masked IL-15-induced hypertrophy in C2C12 cells under steady state condition. However, IL-15 hyperagonist did not induce STAT5 phosphorylation in TNF-α and IFN-γ-pretreated myotubes (Fig. 3e), in which 90 % of endogenous IGF-I was downregulated [59]. Therefore, the scanty amount of IL-15Rβ likely limits the use of IL-15 by the muscle cells.

Although various IL-15 functions in the skeletal muscle were reported previously, some controversial results exist between those in vitro and in vivo studies. In contrast to muscle hypertrophy induced by IL-15 in vitro, muscle atrophy was observed in mice carrying skeletal-muscle-specific IL-15 transgene [18] or receiving systemic infusion of exogenous IL-15 [19]. The muscle atrophy may be contributed by fatal leukemia [60] and metabolic dysregulation [61] induced by overexpression of IL-15 . In addition, recent studies show that exercise endurance increases in skeletal-muscle-specific *Il15*-transgenic mice and reduces in *Il15*^*−/−*^ mice [18, 20], despite that their earlier ex vivo study found no difference in the fatigue index between the EDL or soleus muscle isolated from *Il15*^*−/−*^ mice and skeletal-muscle-specific *Il15*-transgenic mice [21]. Collectively, the inconsistency between the in vitro/ex vivo and in vivo results suggests that the change of muscle mass or exercise endurance in vivo is not caused by the direct effect of IL-15 on the skeletal muscle.

IL-15 has been reported to be involved in a number of autoimmune diseases, including rheumatoid arthritis, inflammatory bowel disease, and multiple sclerosis, in which IL-15 promotes the effector function of cytotoxic CD8^+^ T cells to destroy the target tissues [62]. Considering that TNF-α and IFN-γ induced the expression of IL-15/IL-15Rα and T-cell interacting molecules in skeletal muscle cells, the stimulated muscle cells may directly communicate with T cells. Indeed, we clearly demonstrated in vitro that the stimulated skeletal muscle cells presented antigen and IL-15 to memory-like CD8^+^ T cells and enhanced their effector function. Following induction of autoimmune myositis in vivo, skeletal-muscle-specific *Il15*^*−/−*^ mice showed reduced mononuclear cell infiltration, histopathology score, and expression of inflammatory molecules. Our data together suggest a scenario in which skeletal muscle IL-15 promotes the production of cytotoxic molecules, such as granzyme, by memory CD8^+^ T cells to necrotize muscle cells, which triggers phagocyte recruitment and inflammation. The locally enriched cytokines, TNF-α and IFN-γ (Fig. 6f), may stimulate IL-15 expression by muscle cells to form a feed-forward loop to perpetuate the inflammatory milieu, which contributes to the progression of autoimmune myositis.

## Conclusions

We provide new insights into the function of skeletal muscle IL-15. Rather than being used by the muscle cell itself, the skeletal muscle IL-15 directly promotes the effector function of memory-like CD8^+^ T cells, which facilitates the formation of a pro-inflammatory skeletal muscle microenvironment during myositis progression. Given that IL-15 is not required for muscle growth and regeneration, IL-15 has the potential to be a suitable therapeutic target for autoimmune myositis.

## Additional files

Additional file 1: Table S1. Primer pairs used in this study. Additional file 2: Figure S1. IL-1α/β or LPS induced moderate expression of IL-15/IL-15Rα protein complex in C2C12 myotubes. (**A–C**) C2C12 myotubes were treated with IFN-α (0 or 10 ng/ml), IL-1α/β (0 or 10 ng/ml), or LPS (0, 0.1, or 1 μg/ml) and examined for the expression of *Il15* and *Il15ra* mRNA at indicated time points. Data represent mean ± SEM of triplicates. (**D–F**) The level of IL-15/IL-15Rα complex protein in cell lysate and culture medium after 24-h treatment with IL-1α/β, LPS, or IL-1α were measured by ELISA. Data were pooled from two and three independent experiments. Data are mean ± SEM. **p* < 0.05, ***p* < 0.01, ****p* < 0.001, in comparison to “0” or “con”. Additional file 3: Figure S2. Phenotype analysis of 12-week-old male wt, *Il15* ^*−/−*^, and *Il15ra* ^*−/−*^ mice. (**A**) Body weight. (**B**) Absolute weight and the percentage of body weight (% BW) of heart and gonadal fat pad. (**C**) Absolute weight and the value normalized to tibia bone length (mg/mm) of gastrocnemius muscle (Gas), tibialis anterior muscle (TA), soleus muscle (Sol), and EDL. One symbol represents one mouse. wt (*n =* 14); *Il15* ^*−/−*^ (*n =* 12); *Il15ra* ^*−/−*^ (*n =* 13). Additional file 4: Figure S3. Comparative analysis of wt and *Il15* ^*−/−*^ primary myoblast differentiation. (**A**) Morphology of wt and *Il15* ^*−/−*^ primary myotubes after differentiation for 2 days. The images are representative of at least three independent experiments. (**B**) Fusion index and differentiation index of wt and *Il15* ^*−/−*^ primary myoblast. Fusion index is the percentage of nuclei in MyHC-positive cells among total nuclei. Differentiation index is the percentage of myotubes with indicated number of nuclei among total myotubes. Each index was calculated from at least 24 microscopic fields with each field containing more than 10 myotubes. Data are mean ± SEM. (**C**) Expression of differentiation-related genes in primary muscle cells. Samples were collected after culturing in growth medium for 24 h (GM) and after switching into differentiation medium for 1 and 2 days (day 1 and 2). Data are mean ± SEM. Data are pooled from three independent experiments. Additional file 5: Figure S4. Cardiotoxin-induced muscle regeneration in the TA muscle of wt and *Il15* ^*−/−*^ mice. TA muscle was injected intramuscularly with cardiotoxin (50 μl, 10 μM, Sigma), dissected out, and fixed in formalin at days 5, 10, and 21 after injection. Fixed muscles were embedded in paraffin for histological examination. (**A**) Representative images of TA muscle histology after cardiotoxin injection for 5 (*n =* 4 each genotype), 10 (*n* = 4 each genotype), and 21 (wt *n* = 2; *Il15* ^*−/−*^ *n* = 3) days. Muscle fibrosis was evaluated by Masson’s trichrome staining in the 21-day cardiotoxin-injected samples. (**B**) Expression profiling of regeneration-related genes in the TA muscle injected with cardiotoxin for 5 and 10 days using qPCR. Each group contains four mice. Scale bar = 50 μm. Data are mean ± SEM. **p* < 0.05, ****p* < 0.001. Additional file 6: Figure S5. Compensatory hypertrophy of plantaris muscle. Lower leg soleus and gastrocnemius muscles were removed without damaging the neurovascular supply. Fourteen days after surgery, plantaris muscles were dissected out and weighted. No body weight change was observed during the experiment. The weight of plantaris muscle of mice received surgery or sham surgery were normalized to body weight. One symbol represents one mouse. ****p* < 0.001.

## Abbreviations

*ACTA*: human alpha-skeletal actin
EDL: extensor digitorum longus muscle
Gas: gastrocnemius muscle
grB: granzyme B
IL-15: interleukin 15
IL-15Rα: interleukin 15 receptor alpha
MyHC: myosin heavy chain
Syt7: synaptotagmin VII
TA: tibialis anterior muscle
wt: wild type
γ_c_: common gamma chain
