# Supplementary material for: Skeletal muscle interleukin 15 promotes CD8+ T-cell function and autoimmune myositis
Source: Skelet Muscle. 2015 Sep 28;5:33. doi: 10.1186/s13395-015-0058-2 (PMC4584479; doi:10.1186/s13395-015-0058-2)
Supplement: Additional file 1: Table S1. — Primer pairs used in this study. [file 13395_2015_58_MOESM1_ESM.pdf]

Additional file 1: Table S1. Primer pairs used in this study.

Gene	Primer Bank ID	Forward	Reverse
Il15	6680407a2	TCTCCCTAAAACAGAGGCCAA	TGCAACTGGGATGAAAGTCAC
Il15ra	29029470a2	TGAACTCCAGGGAGAGGTATG	CTAGGGAGGGGTCTCTGATGC
Il15rb	6680427a1	TGGAGCCTGTCCCTCTACG	TCCACATGCAAGAGACATTGG
Il15rg	7305181a1	CTCAGGCAACCAACCTCAC	GCTGGACAACAAATGTCTGGTAG
Irf1	226874845c1	ATGCCAATCACTCGAATGCG	CCTGCTTTGTATCGGCCTGT
Igf1	6754308a3	CGAGGGGCTTTTACTTCAACA	GGGACGGGGACTTCTGAGT
Myh4	9581821a2	AAACCACCTCAGAGTTGTGGA	GTTCCGAAGGTTCCTGATTGC
iNos	6754872a2	ACATCGACCCGTCCACAGTAT	CAGAGGGGTAGGCTTGTCTC
Socs3	6671758a2	AGAGCGGATTCTACTGGAGC	TGGATGCGTAGGTTCTTGGTC
Cd80	31982961a1	ACCCCCAACATAACTGAGTCT	TTCCAACCAAGAGAAGCGAGG
Cd40	24850127a3	TTGTTGACAGCGGTCCATCTA	GCTGGCACAAATCACAGCA
Pdl1	270341382c3	GTCAATGCCCCATACCGCAA	GGCCTGACATATTAGTTCATGCT
B2m	31981890a1	TTCTGGTGCTTGTCTCACTGA	CAGTATGTTCGGCTTCCCATTC
Tap1	239835764c3	CCAACGTGTGCGAGTCCATTA	AGAGTGAGGTACGGTGACCC
Erap1	146149238c3	AGAGCACCTACAGAACACAGG	AGGGCAGGTTCATCAAAGCAG
Ccl2	6755430a1	TTAAAAACCTGGATCGGAACCAA	GCATTAGCTTCAGATTTACGGGT
Ccl5		GTGCCCACGTCAAGGAGTAT	CCCACTTCTTCTCTGGGTTG
Cxcl5	114842397c3	TGCCCTACGGTGGAAGTCATA	TGCATTCCGCTTAGCTTTCTTT
Cxcl9	162287427c1	GGAGTTCGAGGAACCCTAGTG	GGGATTTGTAGTGGATCGTGC
Cxcl10	10946575b1	CCAAGTGCTGCCGTCATTTTC	TCCCTATGGCCCTCATTCTCA
Icam1	21389311a1	GTGATGCTCAGGTATCCATCCA	CACAGTTCTCAAAGCACAGCG
Vcam1	170295822c1	TTGGGAGCCTCAACGGTACT	GCAATCGTTTTGTATTCAGGGGA
Cd4	7304953a1	TCCTAGCTGTCACTCAAGGGA	TCAGAGAACTTCCAGGTGAAGA
Cd8a	126722839c3	ATGGCTTCATCCCACAACAAG	CGTGTCCCTCATGGCAGAA
F4/80	33859546a1	CCCCAGTGTCCTTACAGAGTG	GTGCCCAGAGTGGATGTCT
Il1b	118130747b1	GAAATGCCACCTTTTGACAGTG	CTGGATGCTCTCATCAGGACA
Tnfa	7305585a1	CCCTCACACTCAGATCATCTTCT	GCTACGACGTGGGCTACAG
Ifng	145966741c2	ACAGCAAGGCGAAAAAGGATG	TGGTGGACCACTCGGATGA
Perforin		CCACGGCAGGGTGAAATTC	GGCAGGTCCCTCCAGTGA
MyHC-emb		CTCAAGGAATCCCGGTCCTT	TTCTCGGCAATCTGTTCAGTGA
Ckm	6671762a3	CATCAAGGGTTACACTCTGCC	CCTGCTCCGTCATGCTCTTC
p21		CAGGCGCAGATCCACAGCGA	TCGGGCCTCCTGGAGACAGC
Myog	13654247a3	CAGTACATTGAGCGCCTACAG	GGACCGAACTCCAGTGCAT

Myod	6996932a3	GCTGCCTTCTACGCACCTG	GCCGCTGTAATCCATCATGC
Pax7		TCTTGGTATAAAATGGGACTTGTGTT	CAAAGCAGACAGATTCACAAAAGC
Myf5		TGCCAGTTCTCCCCTTCTGA	CAAACTGGTCCCCAAACTCATC
36B4	6671569a3	TGTTTGACAACGGCAGCATTT	CCGAGGCAACAGTTGGGTA
