# Supplementary figures and images for: Skeletal muscle interleukin 15 promotes CD8+ T-cell function and autoimmune myositis
Source: Skelet Muscle. 2015 Sep 28;5:33. doi: 10.1186/s13395-015-0058-2 (PMC4584479; doi:10.1186/s13395-015-0058-2)

Additional file 2: Figure S1

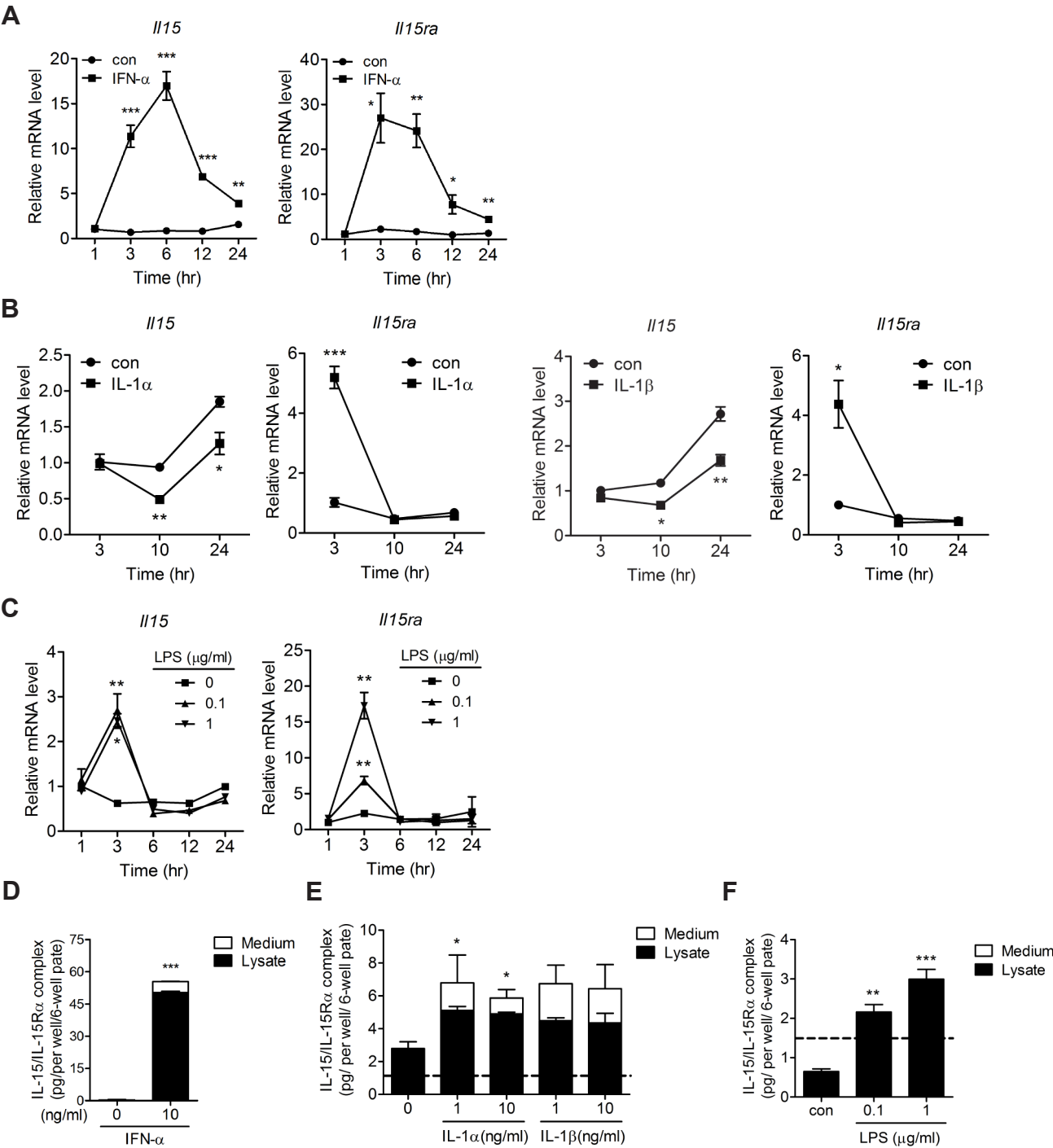

Supplement: Additional file 2: Figure S1. — IL-1α/β or LPS induced moderate expression of IL-15/IL-15Rα protein complex in C2C12 myotubes. (A–C) C2C12 myotubes were treated with IFN-α (0 or 10 ng/ml), IL-1α/β (0 or 10 ng/ml), or LPS (0, 0.1, or 1 μg/ml) and examined for the expression of Il15 and Il15ra mRNA at indicated time points. Data represent mean ± SEM of triplicates. (D–F) The level of IL-15/IL-15Rα complex protein in cell lysate and culture medium after 24-h treatment with IL-1α/β, LPS, or IL-1α were measured by ELISA. Data were pooled from two and three independent experiments. Data are mean ± SEM. *p < 0.05, **p < 0.01, ***p < 0.001, in comparison to “0” or “con”. [file 13395_2015_58_MOESM2_ESM.pdf]

Additional file 3: Figure S2

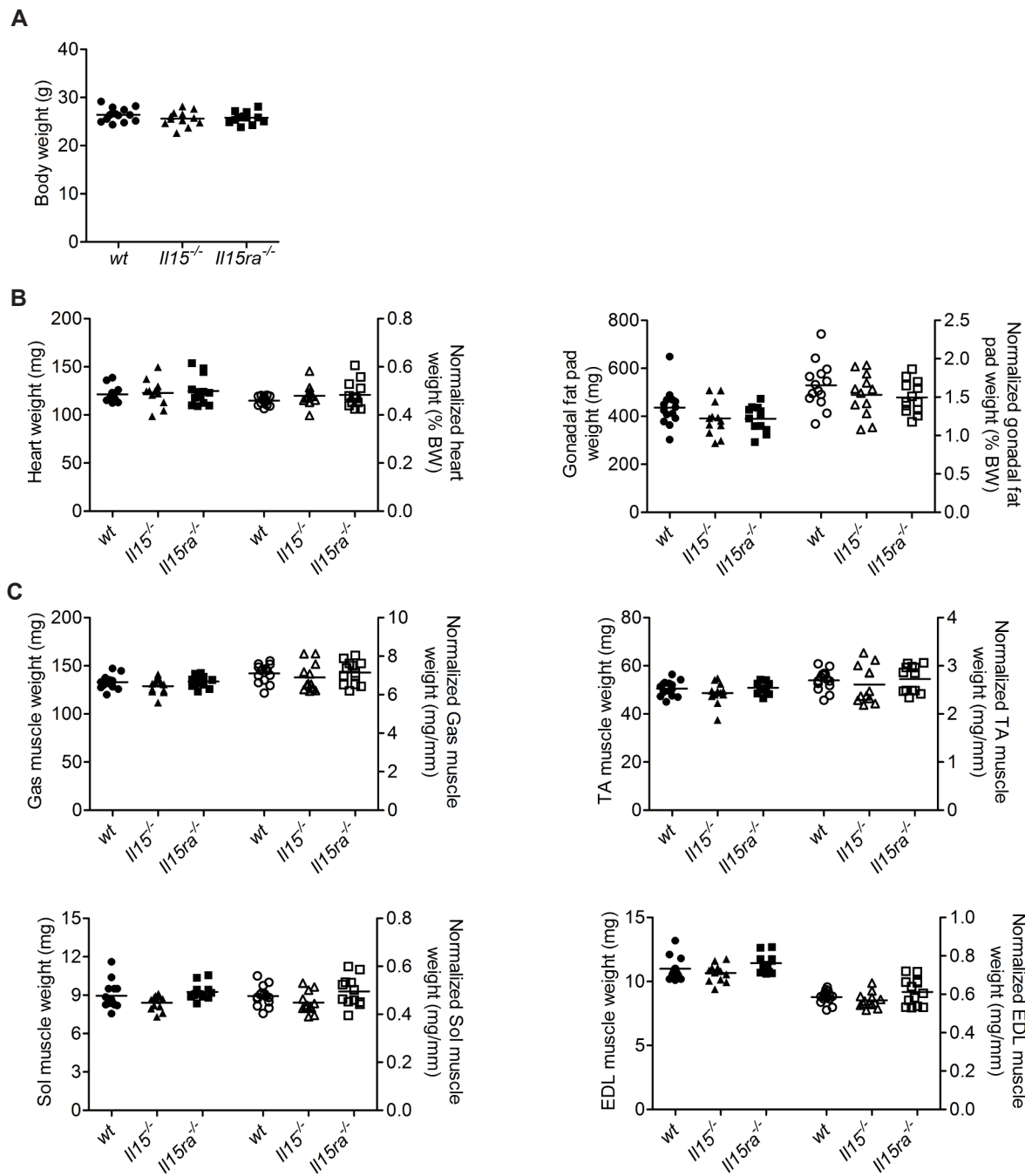

Supplement: Additional file 3: Figure S2. — Phenotype analysis of 12-week-old male wt, Il15 −/−, and Il15ra −/− mice. (A) Body weight. (B) Absolute weight and the percentage of body weight (% BW) of heart and gonadal fat pad. (C) Absolute weight and the value normalized to tibia bone length (mg/mm) of gastrocnemius muscle (Gas), tibialis anterior muscle (TA), soleus muscle (Sol), and EDL. One symbol represents one mouse. wt (n = 14); Il15 −/− (n = 12); Il15ra −/− (n = 13). [file 13395_2015_58_MOESM3_ESM.pdf]

Additional file 4: Figure S3

A

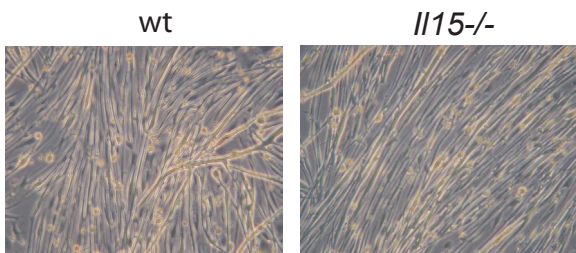

B

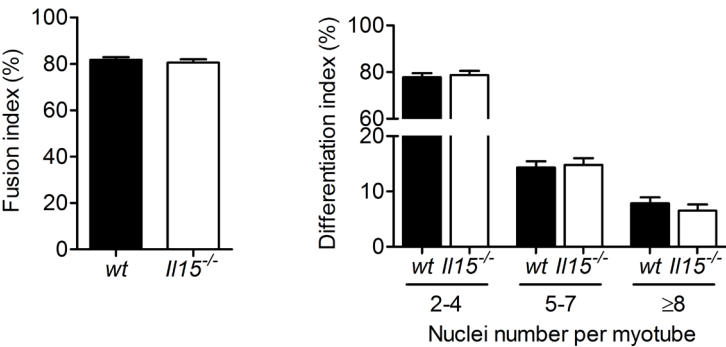

C

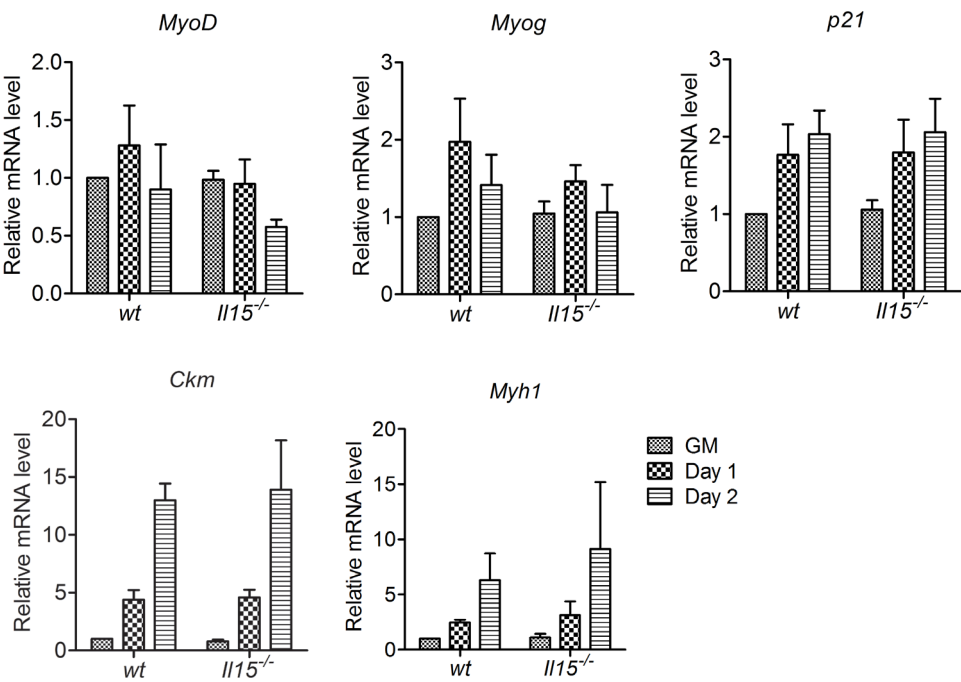

Supplement: Additional file 4: Figure S3. — Comparative analysis of wt and Il15 −/− primary myoblast differentiation. (A) Morphology of wt and Il15 −/− primary myotubes after differentiation for 2 days. The images are representative of at least three independent experiments. (B) Fusion index and differentiation index of wt and Il15 −/− primary myoblast. Fusion index is the percentage of nuclei in MyHC-positive cells among total nuclei. Differentiation index is the percentage of myotubes with indicated number of nuclei among total myotubes. Each index was calculated from at least 24 microscopic fields with each field containing more than 10 myotubes. Data are mean ± SEM. (C) Expression of differentiation-related genes in primary muscle cells. Samples were collected after culturing in growth medium for 24 h (GM) and after switching into differentiation medium for 1 and 2 days (day 1 and 2). Data are mean ± SEM. Data are pooled from three independent experiments. [file 13395_2015_58_MOESM4_ESM.pdf]

Additional file 5: Figure S4

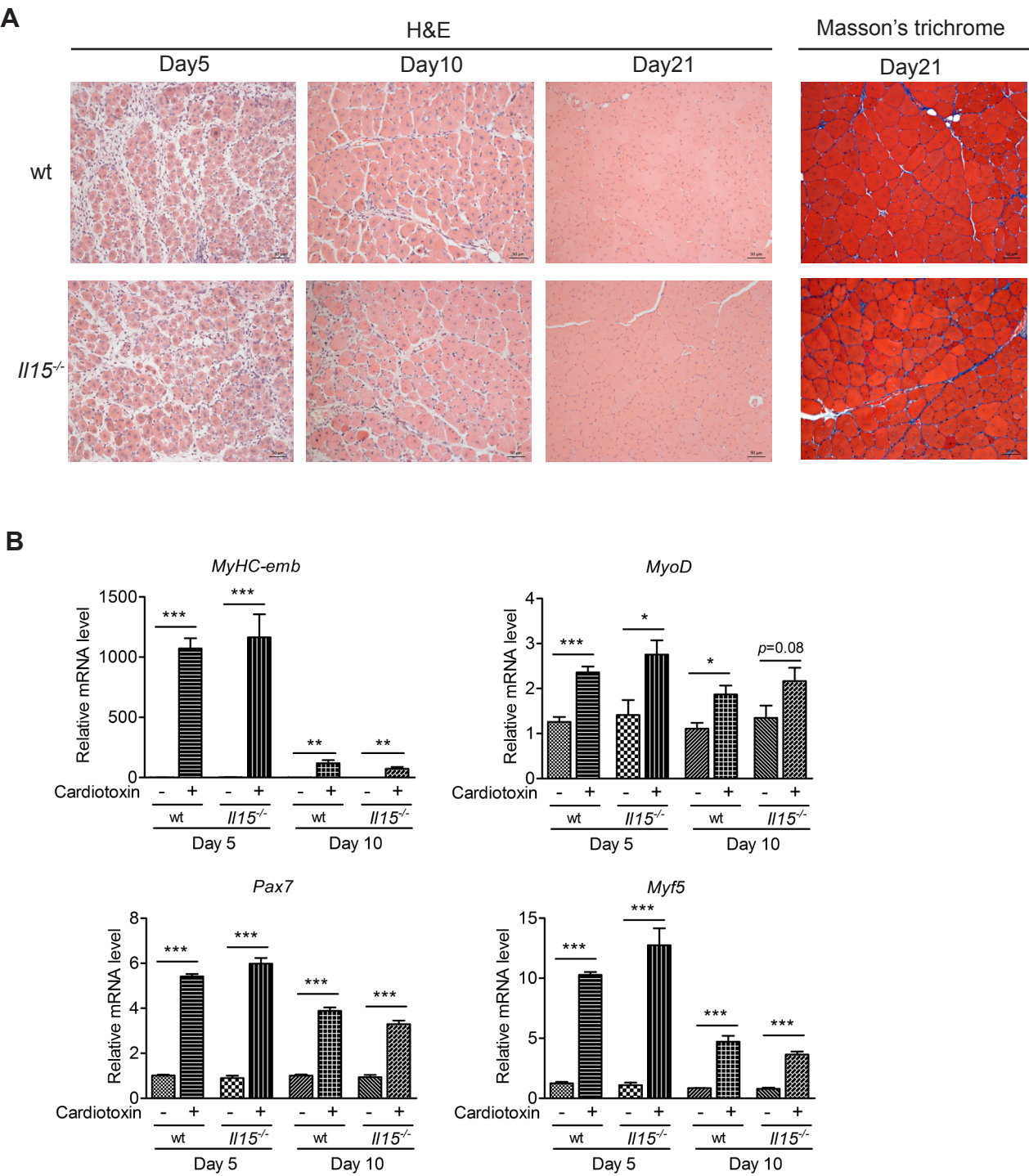

Supplement: Additional file 5: Figure S4. — Cardiotoxin-induced muscle regeneration in the TA muscle of wt and Il15 −/− mice. TA muscle was injected intramuscularly with cardiotoxin (50 μl, 10 μM, Sigma), dissected out, and fixed in formalin at days 5, 10, and 21 after injection. Fixed muscles were embedded in paraffin for histological examination. (A) Representative images of TA muscle histology after cardiotoxin injection for 5 (n = 4 each genotype), 10 (n = 4 each genotype), and 21 (wt n = 2; Il15 −/− n = 3) days. Muscle fibrosis was evaluated by Masson’s trichrome staining in the 21-day cardiotoxin-injected samples. (B) Expression profiling of regeneration-related genes in the TA muscle injected with cardiotoxin for 5 and 10 days using qPCR. Each group contains four mice. Scale bar = 50 μm. Data are mean ± SEM. *p < 0.05, ***p < 0.001. [file 13395_2015_58_MOESM5_ESM.pdf]

## Additional file 6: Figure S5

**A**

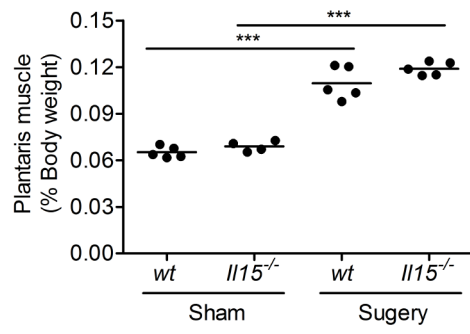

Supplement: Additional file 6: Figure S5. — Compensatory hypertrophy of plantaris muscle. Lower leg soleus and gastrocnemius muscles were removed without damaging the neurovascular supply. Fourteen days after surgery, plantaris muscles were dissected out and weighted. No body weight change was observed during the experiment. The weight of plantaris muscle of mice received surgery or sham surgery were normalized to body weight. One symbol represents one mouse. ***p < 0.001. [file 13395_2015_58_MOESM6_ESM.pdf]
